# Characterization of the *Pseudomonas aeruginosa* metalloendopeptidase, Mep72, a member of the Vfr regulon

**DOI:** 10.1186/1471-2180-13-269

**Published:** 2013-11-27

**Authors:** Aysegul Balyimez, Jane A Colmer-Hamood, Michael San Francisco, Abdul N Hamood

**Affiliations:** 1 Biology Department, Texas Tech University, Lubbock, TX, USA; 2 Department of Immunology and Molecular Microbiology, Texas Tech University Health Sciences Center, Lubbock, TX, USA

## Abstract

**Background:**

*Pseudomonas aeruginosa* Vfr (the virulence factor regulator) enhances *P. aeruginosa* virulence by positively regulating the expression of numerous virulence genes. A previous microarray analysis identified numerous genes positively regulated by Vfr in strain PAK, including the yet uncharacterized *PA2782* and *PA2783*.

**Results:**

In this study, we report the detailed characterization of *PA2783* in the *P. aeruginosa* strain PAO1. RT-PCR analysis confirmed that *PA2782-PA2783* constitute an operon. A mutation in *vfr* significantly reduced the expression of both genes. The predicted protein encoded by *PA2783* contains a typical leader peptide at its amino terminus end as well as metalloendopeptidase and carbohydrate binding motifs at its amino terminus and carboxy terminus regions, respectively. An in-frame *PA2783::phoA* fusion encoded a hybrid protein that was exported to the periplasmic space of *Escherichia coli* and *P. aeruginosa*. In PAO1, the proteolytic activity of the *PA2783*-encoded protein was masked by other *P. aeruginosa* extracellular proteases but an *E. coli* strain carrying a *PA2783* recombinant plasmid produced considerable proteolytic activity. The outer membrane fraction of an *E. coli* strain in which *PA2783* was overexpressed contained specific endopeptidase activity. In the presence of cAMP, purified recombinant Vfr (rVfr) bound to a 98-bp fragment within the *PA2782-PA2783* upstream region that carries a putative Vfr consensus sequence. Through a series of electrophoretic mobility shift assays, we localized rVfr binding to a 33-bp fragment that contains part of the Vfr consensus sequence and a 5-bp imperfect (3/5) inverted repeat at its 3′ and 5′ ends (TGGCG-N_22_-CGCTG). Deletion of either repeat eliminated Vfr binding.

**Conclusions:**

*PA2782* and *PA2783* constitute an operon whose transcription is positively regulated by Vfr. The expression of *PA2783* throughout the growth cycle of *P. aeruginosa* follows a unique pattern. *PA2783* codes for a secreted metalloendopeptidase, which we named Mep72. Mep72, which has metalloendopeptidase and carbohydrate-binding domains, produced proteolytic and endopeptidase activities in *E. coli*. Vfr directly regulates the expression of the *PA2782-mep72* operon by binding to its upstream region. However, unlike other Vfr-targeted genes, Vfr binding does not require an intact Vfr consensus binding sequence.

## Background

*Pseudomonas aeruginosa* is a Gram-negative, opportunistic pathogen that causes acute and chronic infections in immunocompromised hosts, including severely burned patients, individuals with cystic fibrosis, transplant recipients and cancer patients undergoing chemotherapy [1-3]. Virulence of *P. aeruginosa* in these severe infections depends on the production of cell-associated and extracellular virulence factors [1,4,5]. Among the extracellular virulence factors produced by *P. aeruginosa* are the type III secretion system (TTSS), which is a needle-like structure that injects cytotoxins from the cytoplasm of *P. aeruginosa* directly into the cytoplasm of host cells, exotoxin A (ETA), the LasB protease (elastase), LasA, alkaline protease, and phenazines [4-11]. Cell-associated factors are lipopolysaccharide (LPS), the alginate capsule, the flagellum, and the pili [4,5,12]. The production of these factors is controlled by different regulatory proteins, among which is the global regulator Vfr (virulence factor regulator) [13,14]. Vfr, which belongs to the family of cyclic AMP (cAMP) receptor proteins (CRP) and has 90% similarity to the *Escherichia coli* CRP, was originally described as a *P. aeruginosa* factor that is required for the production of ETA and protease IV [15]. Further studies have demonstrated that Vfr activates the transcription of several other virulence genes, such as genes encoding different components of the type III secretion system; as well as the quorum sensing (QS) genes *lasR* and *rhlR*, and *rpoS*, which encodes the stationary phase sigma factor [13,16-18]. Kanack *et al.* showed that Vfr specifically binds to the upstream regions of its target genes [18].

Using microarray analysis, Wolfgang *et al.* identified more than 200 genes that are regulated either positively or negatively by Vfr, including those that encode components of the type III secretion system such as *exoS* and *exsA*[19]. Among the genes whose expression was reduced in the *vfr* mutant compared with its parent strain were *PA2782* and *PA2783*[19]. In this study, we report the characterization of the protein encoded by *PA2783* (PA2783) and a detailed analysis of the regulation of *PA2782* and *PA2783* by Vfr.

## Results

### Vfr regulates the transcription of the *PA2782-PA2783* operon

**Figure 1:**
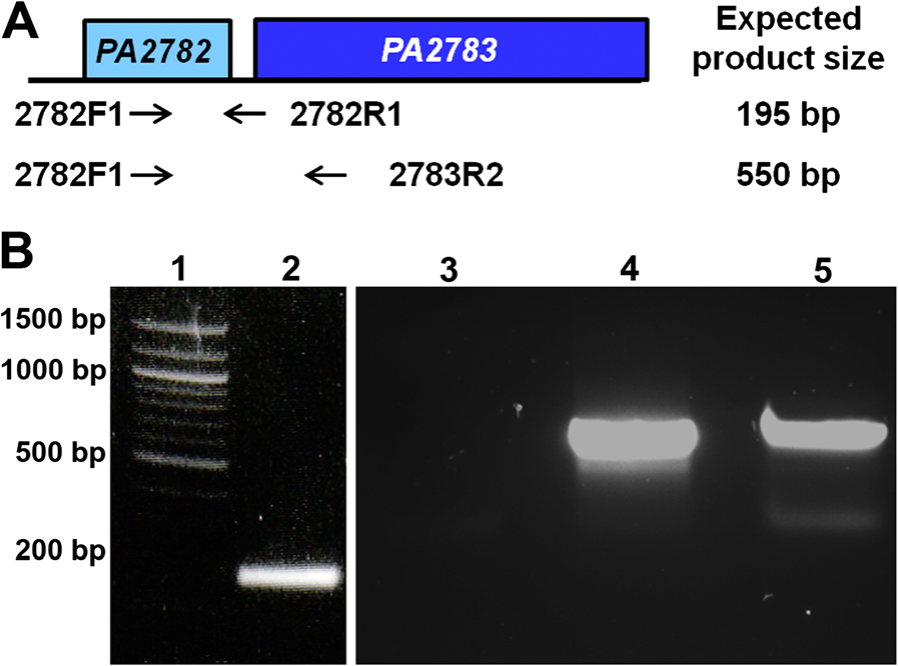
***PA2782 *and *PA2783 *constitute an operon. (A)** Diagram of the two genes showing their relative size, spacing, and direction of transcription (left to right). Location of the primer pairs, 2782F1-2782R1 and 2782F1-2783R2 (black arrows), and the sizes of the expected products are indicated on the diagram. **(B)** PCR products obtained from RT-PCR experiments. Overnight culture of PAO1 was subcultured into fresh LB to a starting OD_600_ of 0.02 and incubated to OD_600_ 0.37. Total RNA was extracted from the cells, purified, and used in reverse transcription reactions to produce cDNA. The cDNA was used as a template in PCR reactions with the primer pairs indicated in **(A)**. PAO1 genomic DNA was extracted and used as a positive control and RNA without reverse transcription was used as a negative control. PCR products were separated on 0.8% agarose and stained with ethidium bromide. Lanes: 1) 100-bp molecular size standard, 2) cDNA plus primers 2782F1-2782R1, 3) RNA without reverse transcriptase plus primers 2782F1-2782R2, 4) genomic DNA plus primers 2782F1-2782R2, 5) cDNA plus primers 2782F1-2783R2.

*PA2782* is located immediately upstream of *PA2783* and the two genes are separated by 78 bp. Computer analyses using the *Pseudomonas* Genome Database suggested that the two genes represent an operon (data not shown) [20]. To confirm this experimentally, we used reverse transcriptase PCR (RT-PCR) and primers corresponding to specific sequences within either *PA2782* alone or within both genes to detect transcripts from PAO1 grown to OD_600_ 0.37 (Figure 1A, Additional file 1). We detected a 550-bp transcript that overlaps the two genes (Figure 1B, lane 5). As a control, we detected a 195-bp transcript produced by two primers corresponding to specific sequences within *PA2782* (Figure 1B, lane 2). As a negative control, the RNA sample was subjected to PCR without reverse transcriptase (Figure 1B, lane 3). As a positive control, we used PAO1 genomic DNA as a template for the 550-bp product (Figure 1B, lane 4).

**Figure 2:**
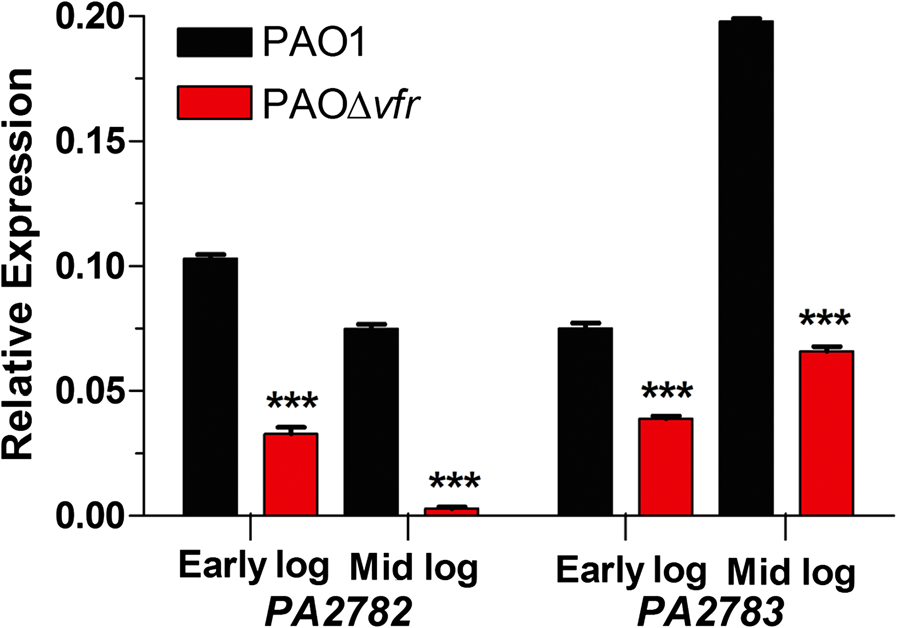
**Vfr regulates the transcription of *PA2782 *and *PA2783 *at early and late stages of growth of PAO1.** PAO1 and PAOΔ*vfr* strains were grown in LB broth overnight and subcultured into fresh LB broth to a starting OD_600_ of 0.02. Cells were harvested at 4 h and 6 h, OD_600_ of 0.37 and 0.79 for PAO1 and 0.41 and 0.89 for PAOΔ*vfr*, respectively, and total RNA was extracted. Levels of *PA2782* and *PA2783* mRNA in each sample were determined by qRT-PCR using specifically-designed primers. Values represent the means of three independent experiments ± SEM. ***P <0.001.

A previous microarray analysis revealed that Vfr regulates the expression of the *P. aeruginosa* genes *PA2782* and *PA2783*[19]. *PA2783* expression was significantly reduced in the *vfr* deletion mutant PAK∆*vfr* compared with its parent strain PAK [19]. While PAK has been extensively studied in lung and corneal infections [21-23], its effects in wound infections, a major emphasis in our laboratory, is less characterized. *P. aeruginosa* strain PAO1 is highly virulent in wound infections, including burn wounds, and has been well-studied in connection with infections in those with cystic fibrosis [24-27]. Therefore, using qRT-PCR, we determined whether Vfr regulates the expression of *PA2782* and *PA2783* in PAO1. We compared the expression of both genes in PAO1 and its *vfr* isogenic mutant PAO∆*vfr* at early (OD_600_ of 0.37 and 0.41) and mid (OD_600_ of 0.79 and 0.89) logarithmic phases of growth. As shown in Figure 2, at both time points and compared with PAO1, the expression of *PA2782* and *PA2783* was significantly reduced in PAO∆*vfr*.

**Figure 3:**
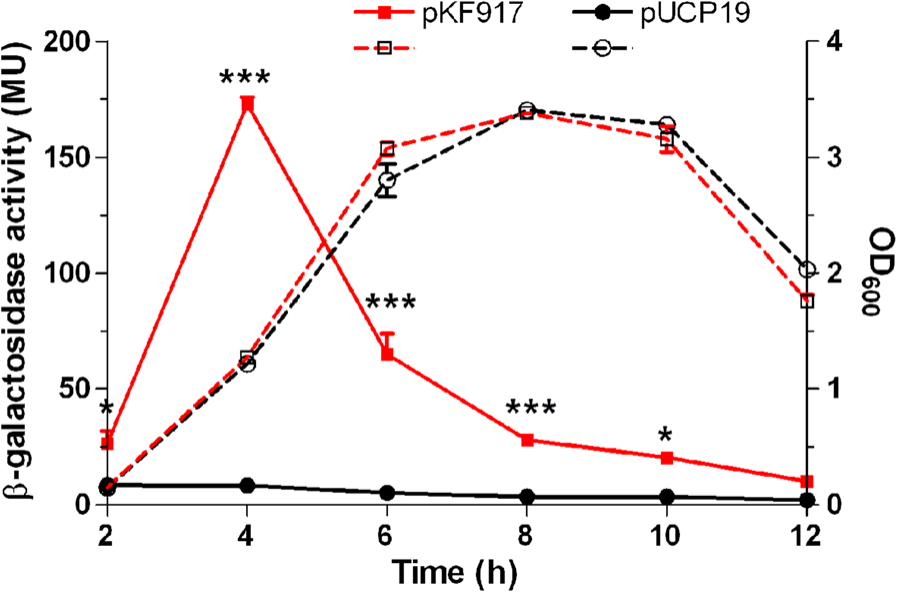
**Vfr regulates *PA2783 *expression throughout the growth cycle of PAO1.** The PAO1 *PA2783* mutant PW5661 carrying either pUCP19 (empty vector) or pKF917, which carries *vfr*, was grown for 12 h. Samples were obtained every 2 h post-inoculation and the level of β-galactosidase activity was determined. Values represent the means of three independent experiments ± SEM. *P <0.05, ***P <0.001.

Due to the presence of functional domains within the predicted protein encoded by *PA2783* (see below), we decided to focus our effort on *PA2783*. We determined the regulation of *PA2783* expression by Vfr throughout the growth cycle of PAO1. This was done using the PAO1 mutant strain PW5661, which carries an in-frame *PA2783*::*lacZ* chromosomal fusion in which the first nine amino acids of the PA2783 protein are fused with the β-galactosidase protein (http://www.gs.washington.edu/labs/manoil/two_allele_August2012.xls) and the *vfr* multicopy plasmid pKF917 (Table 1) [15,28]. Cells were grown in LB broth for 12 h. Samples were obtained every 2 h and the levels of β-galactosidase activity was determined as previously described [29,30]. Compared with PW5661 carrying a vector control (pUCP19), PW5661/pKF917 produced a significantly higher level of *PA2783* expression from 2 h post-inoculation through 10 h, with a sharp peak of expression at 4 h post-inoculation (early to mid-log, OD_600_ 0.15-1.24) (Figure 3). Following this peak, expression of *PA2783* gradually declined towards the 12 h time point (late stationary phase, OD_600_ 2.94-3.22) (Figure 3). This pattern of expression did not result from the effect of pKF917 on the growth of PW5661 since its growth was comparable to that of PW5661 containing the cloning vector (Figure 3). Although in Figures 2 and 3, the time point at which the highest level of *PA2783* expression was detected is different (6 h vs. 4 h post-inoculation), the growth of PAO1 at these two time points is close (OD_600_ of 0.89 for the 6-h time point in Figure 2 and OD_600_ of 1.2 for the 4-h time point in Figure 3). This variation in the growth is possibly due to the presence of a plasmid in PAO1 (pKF917 or pUCP19).

**Table 1:** Strains and plasmids used in this study

**Strains or plasmids**	**Description**	**Source (Reference)**
***Pseudomonas aeruginosa***		
PAO1	Prototrophic strain	[31]
PW5661	*PA2783-*E12::IS*lacZ*/hah; insertion at bp 27 of 1800 in the ORF; Tc^r^	[28]
PAOΔ*vfr* (PAO9001)	*vfr* deletion of PAO1; Gm^r^	[32]
PAO-R1	Δ*lasR*::*tet* derivative of PAO1; Tc^r^	[33]
***Escherichia coli***		
DH5α	Φ80*lac*ZΔM15 *rec*A1 *end*A1 *hsd*R17 (rK–, mK+) *pho*A *sup*E44 λ– *thi*-1 *gyr*A96 *rel*A1	Invitrogen
CC118	F^−^ Δ*(ara-leu)7697 araD139* Δ*lacX74 galE galK* Δ*phoA20 thi rpsE rpoB argE*(Am) *recA1 appR1*	[34]
CC102	F42 *lacI3* zzf-2::Tn*phoA*/CC118	[34]
LMG194	F^−^ Δ*lacX74 galE galK thi rpsL* Δ*phoA* (*PvuII)* Δ*ara714 leu*::Tn*10*	Invitrogen
**Plasmids**		
pUCP19	*E. coli-P. aeruginosa* shuttle vector; Cb^r^	[35]
pKF917	pUCP19 carrying *vfr*; Cb^r^	[15]
pCR™2.1-TOPO®	3.9 kbp TA cloning vector; Cb^r^, Km^r^	Invitrogen
pAB1	pCR2.1-TOPO carrying *PA2783*; Cb^r^ , Km^r^	This study
pAB2	pUCP19 carrying *PA2783* expressed from P_*lac*_; Cb^r^	This study
pAB3	pAB2 carrying a *phoA* fusion; Cb^r^, Km^r^	This study
pBAD/HisC	pBR322-derived expression vector in which cloned genes are expressed from the *ara*BAD promoter (P_BAD_); Cb^r^	Invitrogen
pAB4	pBAD/HisC carrying *PA2783* expressed from P_BAD_; Cb^r^	This study

ORF, open-reading frame; ^r^, resistant; Cb, carbenicillin; Gm, gentamicin; Km, kanamycin; Tc, tetracycline.

The qRT-PCR assay measures the accumulated *PA2783* mRNA within the cell. All available evidence indicates that Vfr is a transcriptional regulator [13,14,18,19]. *PA2783*::*lacZ* is a translational fusion. Thus, the unique pattern of PA2783 expression throughout the growth cycle of PAO1 is likely due to the effect of potential Vfr-independent factors that regulate PA2783 at the translational or post-translational level. The same pattern of expression likely exists in PW5661/pUCP19. However, due to the low level of *PA2783* transcription in this strain, we did not detect the pattern of *PA2783* expression (Figure 3). As pKF917 enhanced *PA2783* transcription, the pattern was detectable (Figure 3).

### The PA2783 protein carries a functional leader sequence

Computer analysis revealed the presence of an export signal within the amino terminus region of the predicted protein encoded by *PA2783* (see below). To examine this possibility experimentally, we first constructed a *PA2783*::*phoA* fusion plasmid. We synthesized an 1807-bp fragment containing the *PA2783* open reading frame (ORF) by PCR and cloned the fragment into pCR2.1-TOPO (Table 1). We then confirmed the presence of the insert in recombinant plasmid pAB1 by DNA sequence analysis (data not shown) (Table 1). The fragment containing *PA2783* was then subcloned into pUCP19 generating recombinant plasmid pAB2 (Table 1). DNA sequence analysis confirmed that, in pAB2, *PA2783* is expressed from the *lac* promoter (data not shown). To determine if PA2783 is exported across the cytoplasmic membrane, pAB2 was transformed into the *E. coli* strain CC102 that carries transposon Tn*phoA* (Table 1). Tn*phoA* mutagenesis was conducted as described in Methods [34]. Tn*phoA* carries the region that codes for the complete alkaline phosphatase protein minus the leader peptide; therefore, an in-frame fusion that provides the protein with a leader peptide would produce functional secreted alkaline phosphatase. We recovered several potential clones including pAB3, which was transformed into the *E. coli* alkaline phosphatase-deficient strain CC118 (Table 1). The resulting transformants produced blue color colonies on XP indicator plates suggesting the presence of functional alkaline phosphatase. DNA sequence analysis confirmed the fusion between the sequences that code for the first 392 aa of PA2783 and the alkaline phosphatase protein (data not shown). To confirm this result, CC118/pAB3 was grown in LB broth for 6 h, the cells were fractionated, and the level of alkaline phosphatase activity within different fractions was determined [34,36]. Alkaline phosphatase activity was detected in the periplasmic and membrane fractions and within the supernatant at a very low level (data not shown). This strongly supports the possibility that PA2783 carries a functional leader peptide.

**Figure 4:**
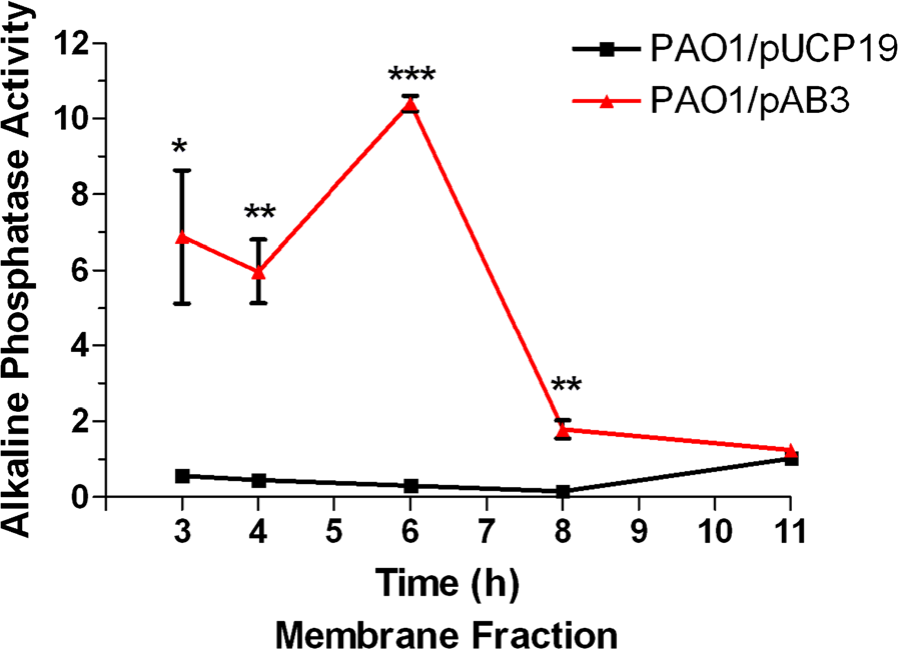
**PA2783 is exported to the outer membrane in PAO1.** Overnight cultures of PAO1 were subcultured in LB broth and grown to the time points indicated on the graph. Cells from 1-ml samples were fractionated and the level of alkaline phosphatase activity in the outer membrane fraction was determined as previously described. Values represent the means of three independent experiments ± SEM. ***P <0.001.

Next, we introduced pAB3 in PAO1 and examined the pattern of *PA2783*::*phoA* expression. PAO1/pAB3 was grown in LB broth for 11 h, samples were obtained every 2 to 3 h, cells were fractionated, and the level of alkaline phosphatase activity was determined. We detected alkaline phosphatase activity in both periplasmic and membrane fractions, with sufficient activity in the membrane fraction to determine levels throughout the growth cycle of PAO1/pAB3 (Figure 4, data not shown). Despite the difference between the *lacZ* and *phoA* fusion analyses in the post-inoculation time points at which we detected certain aspects of *PA2783* regulation, the actual growth (OD_600_) at specific time points (4 h vs. 6 h) was comparable (data not shown) (Figures 3 and 4). The level of alkaline phosphatase activity in PAO1/pAB3 was high at early stages of growth (3- and 4-h time points, which correspond to OD_600_ of 0.3 and 0.5, respectively), peaked at the 6-h time point (OD_600_ of 1.4), and declined over the remaining incubation period (8- and 11-h time points, which correspond to OD_600_ of 2.3 and 2.8, respectively) (Figure 4, data not shown). The level of alkaline phosphatase activity produced by the *PA2783*::*phoA* fusion is significantly lower than the level of β-galactosidase activity produced by the *PA2783*::*lacZ* fusion (Figures 3 and 4). At this time, we do know the reason for the low level of alkaline phosphatase activity.

Unlike the *PA2783*::*lacZ* fusion experiments in which *PA2783* is expressed from the *PA2782-PA2783* promoter in the presence of multiple copies of *vfr* (pKF917), in the *phoA* fusion experiments, *PA2783* is expressed from the *lac* promoter, which is constitutively expressed in *P. aeruginosa*. However, in both experiments, the pattern of *PA2783* expression throughout the growth cycle of PAO1 is comparable (Figures 3 and 4). The enhancement of *PA2783* transcription in each experiment allowed the pattern to be observed. This further supports the possibility that the pattern of *PA2783* expression is produced by the translational or post-translational regulation of PA2783 through Vfr-independent factors.

### Predicted protein PA2783 contains an endopeptidase domain and two carbohydrate binding modules

**Figure 5:**
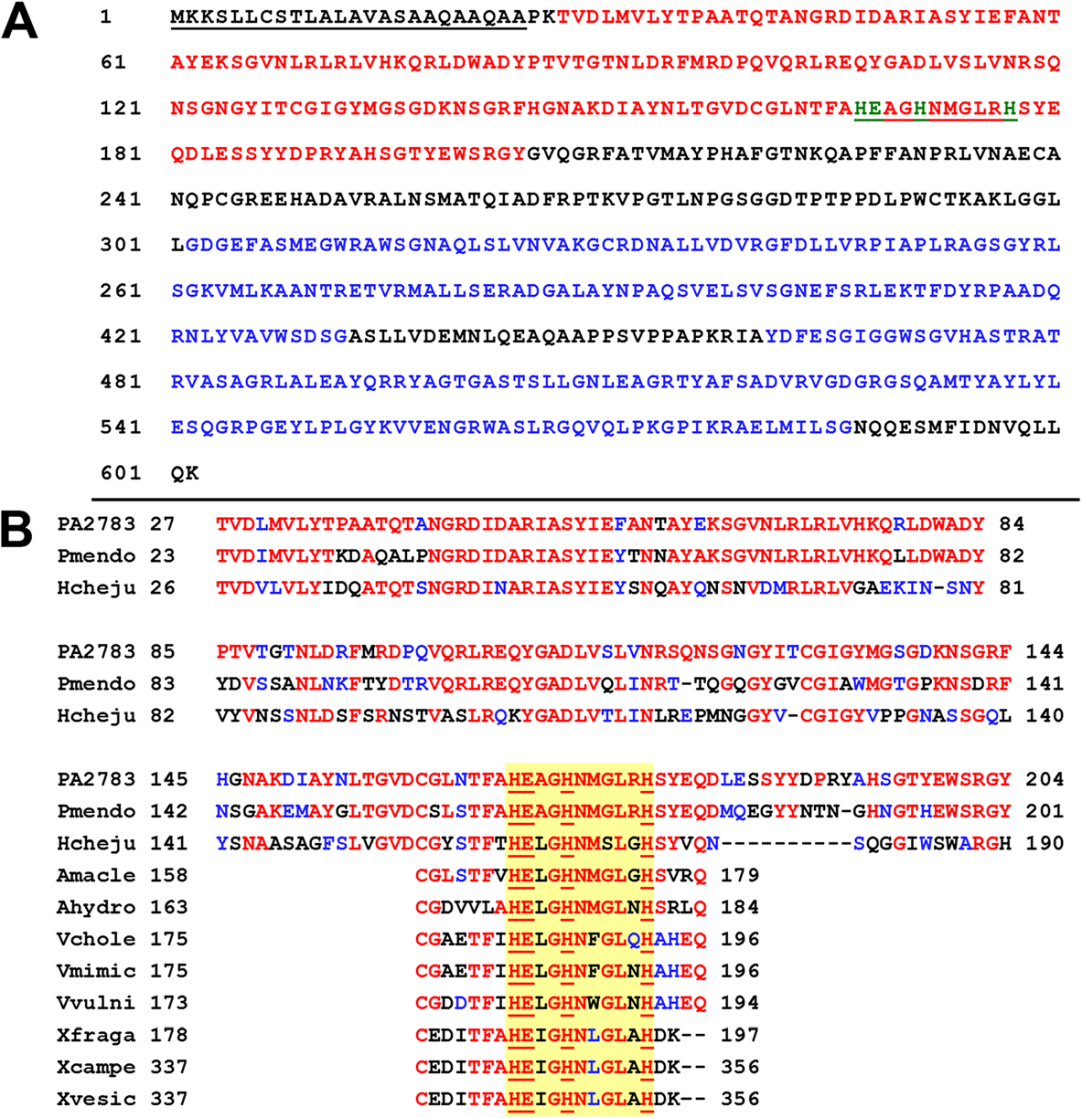
**Characteristics of PA2783 and its homology to other proteins. (A)** Amino acid sequence of the predicted protein encoded by *PA2783*. The 602 aa sequence of PA2783 is shown. The following features are indicated: (1) *Pseudomonas aeruginosa* type I export signal and cleavage site, aa 1 to aa 24, in black and underlined, with the cleavage site at AQA-AP; (2) M72 family peptidase domain, aa 27 to aa 204, in red; (3) the conserved signature **HE**XX**H**XXGLR**H** of M72.001 peptidyl-Asp metallopetidases is underlined; (4) the three conserved histidines (aa 167, 171, and 177), residues for zinc binding, and glutamate (aa 168), the catalytic residue, are in green; (5) two carbohydrate binding modules of the CBM_4_9 family, aa 302 to aa 432 and aa 461 to aa 586, in blue. **(B)** The *P. aeruginosa* predicted PA2783 is homologous to metalloendopeptidases from other bacteria. Interrogation of the non-redundant databases at NCBI (http://www.ncbi.nlm.nih.gov/; accessed 10/18/2013) was done using BLASTP and the Peptidase Database MEROPS (http://merops.sanger.ac.uk/index.shtml; accessed 10/18/2013) was done using BLAST. Identical aa are shown in red, similar aa in blue, and non-similar aa in black. PA2783 is homologous to the *Pseudomonas mendocina* ymp (Pmendo) carbohydrate-binding CenC domain-containing protein and the Ni,Fe-hydrogenase I small subunit of *Hahella chejuensis* KCTC 2396 (Hcheju) across the entire endopeptidase domain. Other proteins contain the **HE**XX**H**XXGXX**H** motif only (highlighted by a yellow box). Amacle, *Alteromonas macleodii*; Ahydro, *Aeromonas hydrophila*; Vchole, *Vibrio cholerae*; Vmimic, *V. mimicus*; Vvulni, *V. vulnificus*; Xfraga, *Xanthomonas fragariae*; Xcampe, *X. campestris*; Xvesic, *X. vesicatoria*. Percentages of aa identity and similarity may be found in Additional file 2.

Computer analysis of the 65-kDa predicted protein encoded by *PA2783* using the SignalP 4.1 Server revealed the presence of a typical *P. aeruginosa* type I export signal and cleavage site at the amino terminus (aa 1 to aa 25) (Figure 5A) (http://www.cbs.dtu.dk/services/SignalP/; accessed 10/18/2013) [37,38]. Additionally, no transmembrane regions were found within the predicted protein (data not shown). The protein contains three specific domains, one at the amino terminus region and two at the carboxyl terminus region (Figure 5A). The amino terminus domain (aa 27 to aa 204) has characteristics of the M72 family of metalloendopeptidases, which include a conserved glutamate catalytic residue (aa 168) and three zinc binding histidine residues (aa 167, 171 and 177) within the motif **HE**XX**H**XXGXX**H** that is common to these proteins (Figure 5A and B; Additional file 2) [39]. The two domains in the carboxy terminus region, located at aa 302–432 and aa 461–586 (Figure 5A), exhibit homology with the carbohydrate (CHO)-binding modules of the CBM_4_9 family of diverse CHO-binding proteins (Additional files 3 and 4) [40]. The strongest overall homology exists between the PA2783 endopeptidase and the *Pseudomonas mendocina* CHO-binding CenC domain-containing protein and the Ni,Fe-hydrogenase I small subunit of *Hahella chejuensis* KCTC 2396 (Figure 5B, Additional file 2). As with PA2783, both proteins contain the metalloendopeptidase domain and the CHO-binding domains I and II. The three proteins have several identical and homologous residues within each domain (Figures 5B; Additional file 2, Additional file 3, Additional file 4).

*PA2782* encodes a putative 22.7-kDa protein of 219 aa that contains no specific motifs, except for the presence of an alanine-rich region within its amino terminus (23 of the first 60 aa), and that has no functional homology with other known proteins (data not shown).

### Characterization of PA2783, a putative metalloendopeptidase

**Figure 6:**
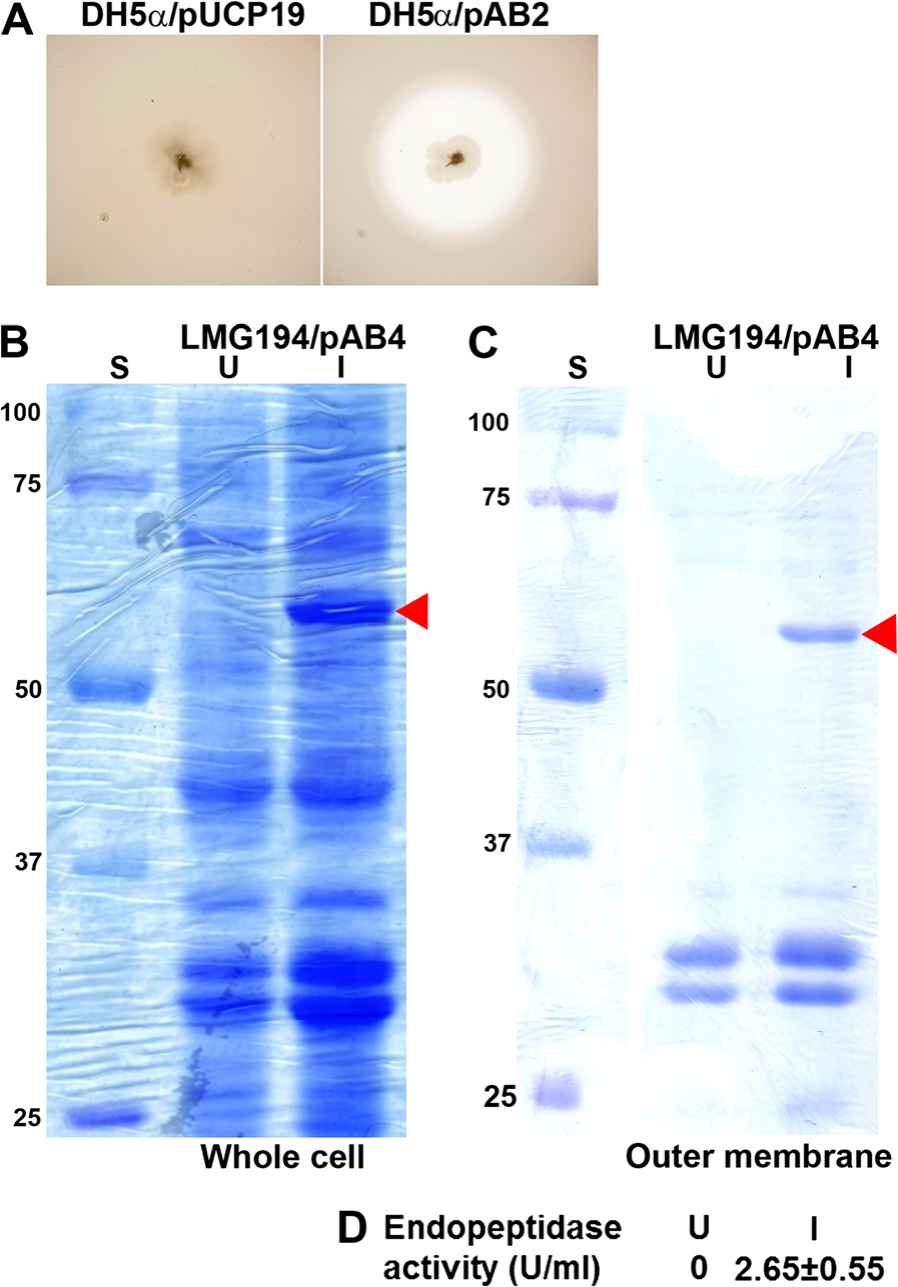
**PA2783 (Mep72) carries proteolytic and endopeptidase activities. (A)** Detection of protease activity produced by PA2783 in *E. coli*. DH5α/pUCP19 (vector control) and DH5α/pAB2 (carrying *PA2783* [*mep72*] under the *lac* promoter) were grown in LB broth and cells were spotted onto skim milk agar plates, incubated at 37°C for 48 h, and observed for zones of clearing. **(B)** Production of recombinant Mep72 (rMep72) in *E. coli*. LMG194/pAB4 (in which *mep72* is expressed from the arabinose promoter) was grown in RM minimal medium supplemented with glucose overnight and subcultured into fresh RM minimal medium. At an OD_600_ of 0.5, 0.002% arabinose was added to induce expression of *mep72* and incubation continued for 5 h. Cells were harvested, lysed, and 10 μg of whole cell lysates were separated by 10% SDS-PAGE, and stained with Coomassie blue. S, molecular mass standards; U, uninduced cells; I, induced cells; arrowhead indicates rMep72. **(C)** Recombinant Mep72 is detected within the outer membrane fraction of *E. coli*. LMG/194/pAB4 was grown as in **(B)**. Cells were harvested and outer membranes were extracted, separated by 10% SDS-PAGE, and stained with Coomassie blue. S, molecular mass standards; U, uninduced cells; I, induced cells; arrowhead indicates rMep72. **(D)** Endopeptidase activity produced by rMep72 was determined as previously described. One unit equals the amount of enzyme sufficient to produce an increase in *A*_520_ of 0.001 per min at 37°C and pH 7.5 (Methods). Values represent the means of three independent experiments ± SEM. U, uninduced cells; I, induced cells.

The predicted protein PA2783 contains all the features of a potential endopeptidase including the putative glutamic acid catalytic residue and the three zinc-binding histidine residues within its amino terminus (Figure 5A) [39]. We tried to assess the proteolytic activity produced by PA2783 using dialyzed brain heart infusion skim milk agar. However, this approach proved unfeasible due to the production by *P. aeruginosa* of several proteases with strong proteolytic activities. Both PAO1/pUCP19 and PAO1/pAB2 produced identical clearing zones of protease activity (data not shown). We faced the same problem when we utilized strain PAO-R1 (Table 1), which produces a considerably reduced level of proteolytic activity due to the mutation of *lasR*[33]. Despite the reduction in the extracellular proteolytic activity of this strain, PAO-R1/pUCP19 and PAO-R1/pAB2 produced identical clearing zones on skim milk agar (data not shown). As an alternative, we assessed the potential proteolytic activity of PA2783 using the *E. coli* strain DH5α (Table 1). Compared with DH5α/pUCP19, which produced no proteolytic zone, DH5α/pAB2 produced a considerable zone of proteolytic activity (Figure 6A) suggesting that the protein is a secreted protease. To examine this possibility, we grew DH5α/pAB2 in LB broth, isolated the supernatant and concentrated it 20X using B15 Minicon concentrators (Millipore, Bedford, MA). However, the concentrated supernatant produced no zone of proteolytic activity on the skim milk agar (data not shown). Whether the growth conditions (skim milk plate vs. LB broth) played a role in the loss or retention of the extracellular protease activity is not known at this time.

Using a previously described endopeptidase assay [41], we tried to determine if at least part of the proteolysis observed on the skim milk plate was due to endopeptidase activity. However, DH5α/pAB2 produced no detectable endopeptidase activity in initial experiments (data not shown). This may be due to the difference in the length of the assays, as the skim milk plates were examined 48 h after inoculation, while the endopeptidase assay results were recorded within 30 min. To remedy this problem, we overproduced recombinant PA2783 (rPA2783) using the pBAD/His expression system (Invitrogen, Carlsbad, CA). The 1807-bp fragment containing *PA2783* was cloned into the expression plasmid pBAD/HisC (Invitrogen) generating pAB4 in which *PA2783* is expressed from the tightly regulated arabinose promoter (Table 1). Plasmid pAB4 was transformed into the *E. coli* expression host LMG194 (Table 1). We grew LMG194/pAB4 in LB broth containing ranges of l-arabinose concentrations (0.2%, 0.02%, 0.002%, and 0.0002%) to an OD_600_ of about 0.5, harvested the cells, and analyzed the protein profile of the lysate using SDS-PAGE. We examined the gels for a unique band that exists in the lysate from induced but not uninduced cultures. We obtained optimum induction using LB broth containing 0.002% arabinose (data not shown). LMG194/pAB4 was grown in RM minimal medium supplemented with glucose overnight and subcultured into fresh RM minimal medium. At an OD_600_ of 0.5, 0.002% arabinose was added to induce expression of *PA2783* and incubation continued for 5 h. Initial examination of total proteins from the whole cell lysate confirmed the overproduction of the protein. As shown in Figure 6B, compared with proteins from the uninduced culture, a unique band that corresponds to the predicted 70.5-kDa recombinant PA2783 protein (rPA2783) was detected in the induced culture. We extracted the band and determined the amino acid sequence of an internal peptide. The sequence matched (100%) that of the predicted protein (data not shown). Using the cold osmotic shock procedure [36,42], we fractionated the cells into supernatant, periplasmic, cytoplasmic, and outer membrane fractions and separated the proteins by SDS-PAGE. Recombinant PA2783 was localized to the membrane fraction (data not shown). As overproduction of foreign proteins in *E. coli* often results in their seclusion in inclusion bodies, which localize with the membrane fraction, we attempted to solubilize rPA2783. Despite trying numerous protocols, we failed to obtain a soluble protein with proteolytic activity. As an alternative, we purified the outer membrane fraction of LMG/pAB4 and examined it for enzymatic activity [41,42]. We detected the 70.5-kDa rPA2783 within the outer membrane preparation of the arabinose-induced cells only (Figure 6C). This was confirmed by amino acid sequence analysis of an internal peptide obtained from the eluted protein (data not shown). Similarly, we detected the endopeptidase activity within the outer membrane of the arabinose-induced cultures only (Figure 6D). These results suggest that *P. aeruginosa PA2783* encodes a membrane-bound 65-kDa protein with endopeptidase activity. We propose the name Mep72 for this protein that belongs to the **m**etallo**e**ndo**p**eptidase family M**72**.001, and *mep72* for the gene encoding it.

### Vfr regulates *mep72* expression by specifically binding to its upstream region

**Figure 7:**
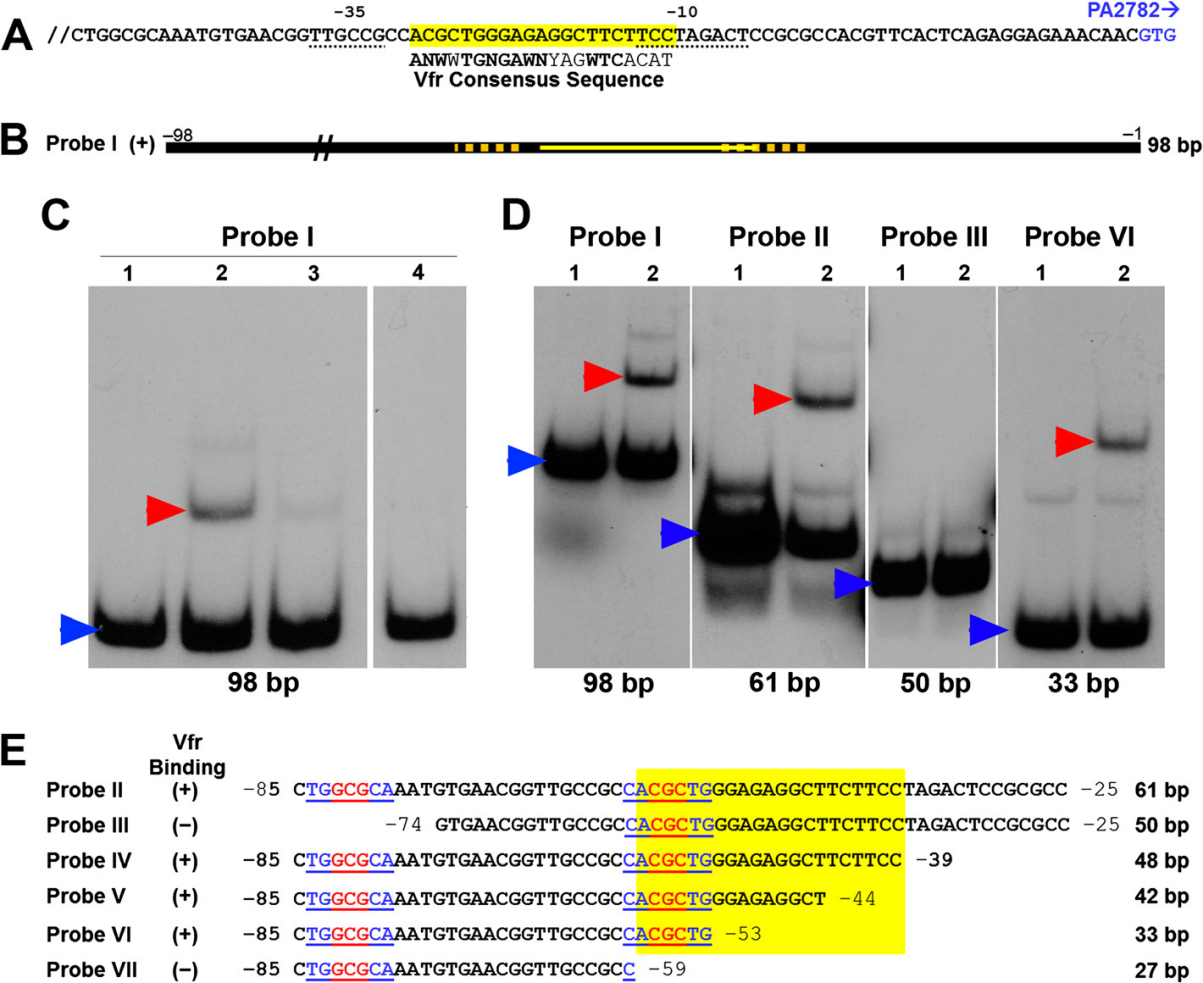
**Vfr specifically binds to the *PA2782-mep72 *upstream region. (A)** Nucleotide sequence of the *PA2782-mep72* upstream region with the putative Vfr binding site indicated by a yellow box. The Vfr consensus sequence is aligned beneath with matching bases in bold; W, purine (A, G); Y, pyrimidine (T, C); N, any base. The −10 and −35 sequences are indicated by dotted lines. The GTG start codon for PA2782 is indicated in blue. **(B)** Diagram of the 98-bp region upstream of *PA2782-mep72* (Probe I); yellow line, Vfr consensus sequence; dotted orange lines, the −10 and −35 sequences. **(C)** Recombinant Vfr binds to the *PA2782-mep72* upstream region. Probe I was prepared by PCR, purified, and radiolabeled. EMSA binding reactions contained approximately 10^5^-10^7^ c.p.m. of labeled probe plus 10 ng purified rVfr (Methods). Samples were separated by 5% SDS-PAGE with 20 mM cAMP added to the running buffer to promote Vfr binding. Lanes: 1) Probe I alone; 2) Probe I plus rVfr; 3) Probe I and rVfr plus excess of unlabelled probe; 4), Probe I plus rVfr (compiled from a separate experiment in which no cAMP was added to the running buffer). Red arrow, Probe I-rVfr complex; blue arrow, unbound Probe I. **(D)** Compiled autoradiographs of gel shift assays using Probes I, II, III, and VI. EMSA were run as described in **(C)** and Methods. Each segment shows probe alone (lane 1) and probe plus 10 ng rVfr (lane 2). Red arrows indicate probe-rVfr complexes; blue arrows, unbound probes. **(E)** Diagram of the nested deletion analysis used to further localize rVfr binding. The matching bases of the 5-bp imperfect inverted repeat (TGGCG/CGCTG) are in red and underlined. These bases are bracketed by two direct repeats (TG-N_3_-CA/TG-N_3_-CA) indicated in blue and underlined.

Vfr is a DNA binding protein that regulates the expression of several genes including *lasR*, *toxR*, *pvdS*, and *ptxR* by binding to the promoter region of these genes [15,16,18,43]. Thus, Vfr may regulate *mep72* expression directly by binding to the upstream region of the *PA2782-mep72* operon. Analysis of the upstream region revealed the presence of a potential Vfr-binding sequence located from −58 to −38 bp 5′ of the PA2782 GTG codon and between the −10 and −35 sequences (Figure 7A) [18]. To determine if Vfr binds to the *PA2782-mep72* upstream region, we conducted electrophoretic mobility shift assays (EMSA). We purified recombinant Vfr (rVfr) as previously described [44]. Since cAMP enhances Vfr binding to its target sequences, we included cAMP in the DNA binding reaction (Methods) [43]. In the presence cAMP, rVfr produced a specific gel shift band with a 98-bp fragment of the upstream region (bp −98 to −1) that carries the intact potential Vfr binding sequence (Probe I) (Figure 7B and C). The binding required cAMP as we failed to detect a binding band when cAMP was eliminated from the binding reaction (Figure 7C).

To localize Vfr binding within the 98-bp fragment, we synthesized two fragments of the *PA2783-mep72* upstream region that were sequentially smaller. A gel shift band was detected using Probe II, 61-bp fragment that included bp −85 to −24 (Figure 7D). However, no gel shift band was detected in EMSA using Probe III, a 50-bp fragment that included bp −74 to −24 (Figure 7D). This suggests that within the 61-bp Probe II, the sequence 5′ of the consensus Vfr binding site is essential for Vfr binding to the upstream region of the *PA2782-mep72* operon.

To further localize the region to which Vfr binds, we conducted nested deletion experiments in which we synthesized several probes that carry nested deletions from the 3′ end of Probe II. The loss of up to 29 bp from the 3′ end (Probes IV, V, and VI) had no effect on Vfr binding (Figure 7D and E). However, the loss of 6 additional bp from probe VI, which deleted the consensus Vfr binding site completely, eliminated Vfr binding (Probe VII) (Figure 7E). Therefore, we localized Vfr binding within the upstream region of *PA2782-mep72* to a 33-bp region that carries only 6 bp of the consensus Vfr binding sequence (Figure 7E). These results suggest that, unlike other Vfr-regulated genes, Vfr binding to the *PA2782-mep72* upstream region does not require the known Vfr consensus sequence.

## Discussion

Experiments described in this study indicate that the *P. aeruginosa* gene *PA2783* encodes a secreted endopeptidase, which we have named Mep72. The predicted protein, which has a typical leader peptide at its amino terminus, belongs to the M72 family of metallopeptidases [39]. According to the MEROPS Peptidase Database, the *P. aeruginosa* Mep72 is a member of the peptidyl-Asp metallopeptidases (M72.001), proteins that degrade aspartate containing substrates by cleaving peptide bonds at the amino side of aspartate or cysteic acid [45]. Additional experiments would be needed to confirm such an activity.

*P. aeruginosa* produces at least three well characterized extracellular proteases/peptidases, LasB, LasA, and PrpL. LasB is a metalloendopeptidase that belongs to the thermolysin (M4) family [39], LasA is a 20-kDa zinc metalloendopeptidase that belongs to the β-lytic endopeptidase family (M23) [39,46], and PrpL is a 27-kDa endopeptidase belonging to the serine endopeptidase family [39,47,48]. Compared with these extracellular proteases, Mep72 has several notable characteristics. First, it is less efficient in proteolytic activity. Neither the loss of the functional gene in *P. aeruginosa* nor the presence of multiple copies of *mep72* (pAB2) in PAO1 or PAO-R1 enhanced the proteolytic activity (data not shown). Second, similar to LasB, LasA, PrpL, and other *P. aeruginosa* proteases, Mep72 is likely to be secreted to the extracellular environment. The lack of transmembrane regions within the predicted protein further supports this suggestion (data not shown). The presence of LasB and other proteases within the PAO1 supernatant prevented us from detecting Mep72 proteolytic activity (data not shown). We were fortunate to detect strong extracellular proteolytic activity in *E. coli* DH5α carrying a *mep72* plasmid (Figure 6A). However, similar to other *P. aeruginosa* proteins, when we overexpressed *mep72* from the pBAD inducible promoter, Mep72 was trapped within the *E. coli* membranes (probably in inclusion bodies) (Figure 6C, D). We plan to produce polyclonal antibodies to the recombinant Mep72 encoded by pAB4 and utilize the antibodies to detect Mep72 within the supernatant of PAO1. Third, unlike LasB, LasA, and PrpL, Mep72 contains additional domains, two CHO-binding modules at the carboxy terminus region (Figure 5A). Whether the CHO-binding and the endopeptidase domains represent two separate functions of Mep72 or are required for a single target is yet to be determined. Fourth, LasB, LasA, and PrpL are among the virulence factors whose production is stringently controlled by the QS system [49]. Since the *P. aeruginosa las* and *rhl* QS systems are controlled by Vfr, the three extracellular proteases are indirectly regulated by Vfr [49]. In contrast, Mep72, which is directly controlled by Vfr, may not be influenced by QS systems. Through several preliminary experiments, we ruled out the possibility that *mep72* expression is regulated by either the *las* or the *rhl* system (data not shown). Fifth, unlike other proteases, the impact of Mep72 on *P. aeruginosa* virulence is not defined yet. The loss of functional Mep72 in PAO1 did not impact the production of several virulence factors including LasB, LasA, pyocyanin, or pyoverdine (data not shown). Additionally, preliminary analysis using the murine model of thermal injury showed that the *in vivo* virulence of PW5661 is comparable to that of its parent strain (data not shown).

The first such endopeptidase enzyme described was isolated from *Pseudomonas fragi*, a pyschrotrophic, proteolytic organism that causes meat spoilage by producing a single extracellular neutral protease, endoproteinase Asp-N, at lower temperatures [50,51]. As Mep72 has amino acid identity with the *P. fragi* protein in the endopeptidase region (data not shown), and since *P. aeruginosa* grows at 10°C, we examined the proteolytic activity of Mep72 at this temperature. At this temperature, Mep72 activity would not be masked by other *P. aeruginosa* extracellular proteases, which are activated at 37°C. However, we did not detect any difference in their proteolytic zones. The two CHO-binding domains carried by Mep72 belong to the CBM_4_9 family. Proteins in this family are important for very diverse CHO metabolic processes including enzymatic degradation of oligosaccharides, cellulase activity and hydrolase activity by acting on glycosyl bonds [40,52,53]. Whether the CBM_4_9 domain in Mep72 plays a role in *P. aeruginosa* binding to the alveolar mucus during lung infections is not known.

All available evidence, including data provided in this study, suggests that Vfr is a DNA-binding transcriptional regulator [13,14,18,19] (Figures 2 and 7). Using qRT-PCR, we also detected transcriptional regulation of *mep72* expression by Vfr (Figure 2). Additionally, one of the unique features of *mep72* is its pattern of expression throughout the growth cycle of PAO1, which we detected with both *lacZ* and *phoA* translational fusions (Figures 3 and 4). In these experiments, *mep72* expression was enhanced by the presence of multiple copies of *vfr* (*lacZ*) or expression the *lac* promoter, which is constitutively expressed in *P. aeruginosa* (*phoA*). The same pattern likely exists in PAO1 and PW5661 carrying pUCP19 (vector control); however, due to the low level of *mep72* expression, we did not detect it. These results strongly suggest that the unique pattern of *mep72* expression is due to the effect of Vfr-independent translational/post-translational regulation.

This pattern of expression is not a feature of the Vfr regulon. Many genes of the Vfr regulon including *lasB*, *lasA*, *lasR* are part of the quorum sensing system and as such, expression is induced at later rather than earlier stages of growth [16,54]. The significance of this pattern of expression is not known at this time. However, during our analysis of the *P. aeruginosa* global regulator PtxR (using *ptxR-lacZ* transcriptional fusions), we previously reported a pattern of expression that mimics that of *PA2782-mep72*[55]. The expression of one of the *ptxR*-promoter nested deletions reached a peak at early stage of growth, sharply declined after that, and continued a low level of expression toward the end of growth cycle [55]. Similar to *mep72*, Vfr binds to the *ptxR* upstream and directly regulates *ptxR* expression [43].

Through the examination of the promoter regions of genes regulated by Vfr including *lasR*, *toxA*, *pvdS*, *prpL*, and *algD*, Kanack *et al.* developed a 21-bp Vfr binding consensus sequence that consist of two halves and contain several conserved nucleotides within each half [18]. Experimental evidence revealed that changing one or more of these conserved nucleotides within the *lasR* or *fleQ* promoters affected the expression of these genes and their regulation by Vfr [16,18,44]. Our current analysis confirmed that Vfr specifically binds to the *PA2782-mep72* promoter (Figure 7C). As with other Vfr-regulated genes, Vfr binding to the *PA2782-mep72* promoter is cAMP dependent (Figure 7C). However, in contrast to all previously identified Vfr binding sites, the potential Vfr binding region within *PA2782-mep72* does not contain the intact Vfr consensus sequence (Figure 7D and E). Rather, we localized Vfr binding within the *PA2782-mep72* promoter to a 33-bp sequence (probe VI), which contains only 6 bp from the left half of the Vfr consensus sequence (Figure 7E). Careful examination of the sequence revealed the presence of a 5-bp imperfect inverted repeat, with two bp mismatch (**underscored**), at either end of the 33-bp sequence: **TG**GCG-N_22_-CGC**TG** (Figure 7E). Compromising either of the repeats eliminated Vfr binding (Figure 7D and E). Thus, this sequence may constitute an alternative Vfr binding site. The TGGCG-N_22_-CGCTG sequence overlaps the −35 region (Figure 7E). Additionally, the 33-bp sequence contains two direct repeats (TG/TG and CA/CA) (Figure 7E). Furthermore, the 33-bp sequence contains another imperfect (7/9) inverted repeat consisting of 9 bp, TGGCGCAAA-N_9_-TTGCCGCCA. Probe VII, which lost the ability to bind Vfr, lacks only one bp (A) from the right side of this repeat (Figure 7E). Further analysis including DNA foot printing experiments will be done to determine the exact sequence to which Vfr binds.

## Conclusions

*PA2782* and *PA2783* constitute an operon whose transcription is positively regulated by Vfr. The expression of *PA2783* throughout the growth cycle of *P. aeruginosa* follows a unique pattern. *PA2783* codes for a secreted metalloendopeptidase, which we named Mep72. Mep72, which has metalloendopeptidase and carbohydrate-binding domains, produced proteolytic and endopeptidase activities in *E. coli*. Vfr directly regulates the expression of the *PA2782-mep72* operon by binding to its upstream region. However, unlike other Vfr-targeted genes, Vfr binding does not require an intact Vfr consensus binding sequence.

## Methods

### Strains, plasmids, and general growth conditions

Bacterial strains and plasmids used in this study are listed in Table 1. For routine growth, strains were grown in Luria-Bertani (LB) broth [29]. Antibiotics were used at the following concentrations as appropriate: for *E. coli*, 100 μg carbenicillin/ml and/or 50 μg kanamycin/ml; for *P. aeruginosa*, 300 μg carbenicillin/ml, 60 μg gentamicin/ml, 300 μg kanamycin/ml, or 50 μg tetracycline/ml.

### General DNA techniques

Plasmid DNA extraction was performed using the Wizard Plus MiniPreps DNA Purification system and genomic DNA was extracted from PAO using the Wizard Genomic DNA Purification kit (Promega, Madison, WI). Restriction digestion, ligation and transformation of *E. coli* were done as described [56]. Plasmids were introduced into *P. aeruginosa* by electroporation [57].

### Construction of cloning and expression plasmids

An 1807-bp PAO1 chromosomal fragment containing the *PA2783* ORF was amplified by PCR using primers PA2783orf-F/PA2783orf-R (see Additional file 1). The PCR product was cloned into pCR2.1-TOPO (Invitrogen, Carlsbad, CA) generating plasmid pAB1. An 1827-bp fragment carrying *PA2783* was excised from the pAB1 plasmid by *EcoRI* digestion and ligated into the *EcoRI* site of the *E. coli-Pseudomonas* shuttle vector pUCP19 to generate plasmid pAB2. Overexpression of *PA2783* to produce rPA2783 (rMep72) was done as follows: the 1827-bp *EcoRI* fragment carrying *PA2783* was excised from pAB1 and ligated into the pBAD/HisC expression vector (Invitrogen) to produce the plasmid pAB4. Construction of plasmids was confirmed by restriction digestion.

### Quantitative reverse transcriptase PCR (qRT-PCR) and RT-PCR

Overnight cultures of *P. aeruginosa* strains PAO1 and PAO1∆*vfr* were subcultured in LB broth to an OD_600_ of 0.02 and grown for up to 6 h at 37°C. Cultures were harvested at early log phase of growth (OD_600_ 0.37-0.41) and mid log phase (OD_600_ 0.79-0.89). Cultures were mixed with twice the volume of RNAprotect Bacteria Reagent (QIAGEN, Valencia, CA) for 5 min at room temperature and the cells were pelleted. Pelleted cells were lysed using lysozyme and proteinase K for 15 min at room temperature, and then the total RNA was extracted using the RNeasy Mini Kit (QIAGEN) according to the manufacturer’s instructions. To remove genomic DNA, the RNA solution was treated with the RNase-free DNase Set (QIAGEN). RNA was purified from DNase by the RNA cleanup protocol (QIAGEN) with an additional on column DNase treatment to eliminate any remaining traces of genomic DNA. RNA was quantified by NanoDrop® spectrophotometer (NanoDrop Products, Wilmington, DE).

cDNA was synthesized from the extracted RNA using the QuantiTech Reverse Transcription Kit (QIAGEN). For qRT-PCR, a 200-ng aliquot of cDNA and 250 nM of specific primer (see Additional file 1) were mixed with SYBR Green PCR Master Mix (Life Technologies, Carlsbad, CA). Three independent biological replicates were used for RNA extraction. Additionally, each PCR reaction was set up in triplicate. The 30S ribosomal RNA gene *rpsL* was used as an internal standard to normalize the quantity of cDNA in different samples [58]. Gene expression analysis was done using StepOne Plus software version 2.2.2 (Life Technologies). For RT-PCR, PCR was performed using the prepared cDNA and specific primers to amplify regions of *PA2782*, and *PA2782*-*PA2783* (see Additional file 1). As a positive control, genomic DNA extracted from PAO1 was amplified by PCR using the primers for *PA2782-PA2783*. PCR extension was conducted at temperatures appropriate for each primer. To exclude DNA contamination, each RNA sample was subjected to PCR without reverse transcriptase. The products were examined using 0.8% agarose gel electrophoresis.

### Tn*phoA* mutagenesis

This was done using the previously described method by Boquet *et al.*[34]. Plasmid pAB2 that carries *PA2783* was transformed into *E. coli* strain CC102 that carries the F’ factor, F42 *lacI3* zzf::Tn*phoA*[34]. The transformants were selected on LB agar plates containing carbenicillin and kanamycin. Individual colonies were grown in LB broth, diluted and spread on LB agar plates containing carbenicillin, kanamycin (300 μg/ml), and chromogenic alkaline phosphatase substrate 5-bromo-4-chloro-3-indolyl phosphate (XP) (40 μg/ml) (Sigma Aldrich). The high kanamycin concentration is essential to enrich for cells in which the Tn*phoA* transposon has inserted in pAB2. Blue color colonies indicative of alkaline phosphatase activity were streaked on the XP plates to confirm the alkaline phosphatase production phenotype. Additionally, plasmid DNA was extracted from these colonies and transformed into the *E. coli* alkaline phosphatase deficient strain CC118. We confirmed the in-frame *PA2783*::*phoA* fusion by DNA sequence analysis using an appropriate primer (see Additional file 1).

### Cellular fractionation

*E. coli* cells were fractionated using the cold shock osmotic procedure as described by Koshland and Botstein and Lee *et al.*[36,42]. Fractionation of *P. aeruginosa* was conducted according to the procedure described by Cheng *et al.*[59].

### Overexpression of rPA2783 (rMep72) and outer membrane preparation

Plasmid pAB4 was transformed into the *E. coli* strain LMG194 and transformants were selected on LB agar with carbenicillin. Transformants were grown for 16 h at 37°C in RM minimal medium (Invitrogen) that was supplemented with 0.2% glucose and carbenicillin. The culture was then inoculated in fresh RM medium (1:100), and incubation was continued at 37°C. At an OD_600_ of 0.5, l-arabinose was added at a concentration of 0.002% and the incubation continued for an additional 5 h. Cells were harvested by centrifugation at 14,000 × *g* for 10 min. Pelleted cells were lysed using Biospec bead beater (Biospec, Bartlesville, OK), and the outer membrane fraction was prepared as previously described with slight modifications [42].

Briefly, pelleted cells were washed with 10 mM phosphate buffer (pH 7.0) and disrupted using bead beater (Biospec) using 1 min burst and 1 min rest three times at 4°C. Unbroken cells were removed by centrifugation at 5,000 × *g* for 10 min at 4°C using Beckman JA20 rotor. The inner membrane was then dissolved by adding 1% lauryl sarcosyl (Sigma Aldrich, St. Louis, MO) and samples were centrifuged at 100,000 × *g* for 1 h. The resulting outer membrane pellet was resuspended in 10 mM phosphate buffer (pH 7.0) and analyzed on 10% SDS-PAGE.

### Electrophoretic mobility shift assays

DNA fragments containing different regions of the *PA2782-mepA* upstream region were synthesized by PCR (see Additional file 1 for specific primers used to synthesize the probes). PCR products were purified from 0.8% agarose gels using the Qiaex II Gel Extraction Kit (QIAGEN). Purified DNA fragments were end-labeled with [γ-^32^P] ATP using T4 polynucleotide kinase [56].

EMSA were performed as described by Ferrell *et al.* with minor modifications [43]. Binding reactions were set up in 25 μl of DNA-binding buffer (10 mM Tris/HCl, pH 7.4, 1 mM EDTA, 10 mM KCl, 1 mM DTT, 5% glycerol and 20 mM cAMP plus 50 mg BSA and 5 mg poly(dI-dC)/ml binding buffer. Each reaction contained 10 ng of purified Vfr and 10^5^–10^7^ c.p.m. of radiolabeled probe. Reactions were incubated for 30 min at room temperature and separated by 5% SDS-PAGE. To promote Vfr binding, 20 mM cAMP was added to the buffer in the upper chamber. Gels were dried and exposed to x-ray film.

### Enzyme assays

The level of β-galactosidase activity was determined as previously described [29,30]. The level of alkaline phosphatase activity within different fractions of *E. coli* and *P. aeruginosa* was determined as previously described [34].

The skim milk agar protease assay was performed using dialyzed brain heart infusion (DBHI) skim milk agar plates prepared as previously described [60]. Each plate was stab-inoculated with either DH5α/pUCP19 or DH5α/pAB2. The plates were incubated at 37°C for 48 h and the diameter of the proteolysis zone around the colonies was measured.

Metalloendopeptidase activity within outer membrane fractions of *E. coli* LMG194 strain containing pAB4 was determined using the modified method of Ensign and Wolfe [41]. Azocoll (2%) in 50 mM Tris buffer pH 7.5 was mixed with 200 μl of outer membrane fraction obtained from either induced (0.002% l-arabinose) or non-induced *E. coli* cultures. Reactions were incubated at 37°C for 30 min, and the absorbance was measured at 520 nm. One unit was defined as the amount of enzyme that releases a sufficient amount of azo dye from azocoll substrate to produce an increase in *A*_520_ of 0.001 per min at 37°C, pH 7.5.

### Murine model of thermal injury

The experiments were conducted as previously described [61]. Animals were treated in accordance with Protocol 96020 approved by the Institutional Animal Care and Use Committee at Texas Tech University Health Sciences Center in Lubbock, TX.

### Statistical analyses

Statistical analyses were done using GraphPad InStat 3.06 (GraphPad Software, San Diego, CA). One-way ANOVA with the Tukey-Kramer multiple comparisons post-test was used to determine significant differences across time. The two-tailed *t*-test was used to compare pairs of strains containing different plasmids.

## Competing interests

The authors declare they have no competing interests.

## Authors’ contributions

AB designed portions of the study, conducted all the experiments, and wrote the manuscript. JACH analyzed and interpreted data and critically revised the manuscript. MSF participated in data analysis. ANH coordinated the project, designed portions of the study, and helped draft and revise the manuscript. All authors have read and approved the final manuscript.

## Supplementary Material

Additional file 1 Oligonucleotides used in this study. Click here for file

Additional file 2 Amino acid homology of the predicted PA2783 protein endopeptidase domain with other bacterial proteins. Click here for file

Additional file 3 **The predicted PA2783 protein carries two carbohydrate-binding modules.** Interrogation of the non-redundant databases at NCBI (http://www.ncbi.nlm.nih.gov/; accessed 06/19/2013) using BLASTP revealed homology with the CBM_4_9 family (Cdd:pfam02018) of diverse CHO-binding proteins. CHO-binding domain I (A) and domain II (B), have different aa sequences but both were strongly homologous to the two CHO-binding modules of the *Pseudomonas mendocina* (Pmendo) carbohydrate-binding CenC domain-containing protein and the Ni,Fe-hydrogenase I small subunit of *Hahella chejuensis* (Hcheju). For the pfam, identical aa are indicated by * and similar aa by ^; for bacterial proteins, identical aa are shown in red, similar aa in blue, and non-similar aa in black; Pmucil, *Paenibacillus mucilaginosus*; Clen-1 and Clen-2, *Cellulosilyticum lentocellum* CHO-binding domains I and II. Percentages of aa identity and similarity are shown in Additional file 4. Click here for file

Additional file 4 Amino acid homology of the predicted PA2783 protein carbohydrate-binding domains I and II with other bacterial proteins. Click here for file
