# Supplementary material for: Characterization of the Pseudomonas aeruginosa metalloendopeptidase, Mep72, a member of the Vfr regulon
Source: BMC Microbiol. 2013 Nov 27;13:269. doi: 10.1186/1471-2180-13-269 (PMC4222646; doi:10.1186/1471-2180-13-269)
Supplement: Additional file 1 — Oligonucleotides used in this study. [file 1471-2180-13-269-S1.pdf]

## Additional file 1 - Oligonucleotides used in this study

| Primer Pairs | Forward (5' - 3') | Reverse (5' - 3') |
|---|---|---|
| **Gene expression** | | |
| PA2782F/R | GCCAGCACCCATAACAGC | GGCGATGTCGTTCTCGTC |
| PA2783F/R | AATCCCTGCTCTGCTCCAC | GTTGACGCCGCTCTTCTC |
| *rpsL*F/R | CCACCACGCCGAAAAA | CGAACGACCCTGCTTACG |
| **RT-PCR to confirm operon** | | |
| 2782F1/2782R1 | GCCAGCACCCATAACAGC | GGCGATGTCGTTCTCGTC |
| 2782F1/2783R2 | GCCAGCACCCATAACAGC | GTTGACGCCGCTCTTCTC |
| **Cloning *PA2783*** | | |
| PA2783orf-F/ PA2783orf-R | CGAACACAGGCAACCCATGAAGAA | ACGAGGGAAACCGGAGTGATCATA |
| **Sequencing of Tn*phoA* mutants** | | |
| Tn*phoA*-seq | AGCCCGGTTTTCCAGAACAGG | |
| **Probes for electophoretic mobility shift assays** | | |
| Probe I-F/R | GCCGGCGCGCGGCCTGGCGCAAAT | GTTGTTTCTCCTCTGAGTGAACG |
| Probe II-F/R | CTGGCGCAAATGTGA | TGGCGCGGAGTCTAGGA |
| Probe III-F/R | GTGAACGGTTGCCGC | TGGCGCGGAGTCTAGGA |
| Probe IV-F/R | CTGGCGCAAATGTGA | GAAGAAGCCTCTCCCAGCG |
| Probe V-F/R | CTGGCGCAAATGTGA | AGCCTCTCCCAGCGT |
| Probe VI-F/R | CTGGCGCAAATGTGA | CAGCGTCCGCCGAACCGT |
| Probe VII-F/R | CTGGCGCAAATGTGA | GGCGGCAACCGTTCACATT |
