# Supplementary material for: Characterization of the Pseudomonas aeruginosa metalloendopeptidase, Mep72, a member of the Vfr regulon
Source: BMC Microbiol. 2013 Nov 27;13:269. doi: 10.1186/1471-2180-13-269 (PMC4222646; doi:10.1186/1471-2180-13-269)
Supplement: Additional file 2 — Amino acid homology of the predicted PA2783 protein endopeptidase domain with other bacterial proteins. [file 1471-2180-13-269-S2.pdf]

Additional file 2 - Amino acid homology of the predicted PA2783 protein endopeptidase domain with other bacterial proteins

Organism<br>Accession Number	Protein	Identity (%)	Similarity (%)	Gaps (%)
Homology across entire metalloendopeptidase domain				
Pseudomonas mendocina<br>YP_001189641.1 ymp	CHO-binding CenC domain-containing protein	124/178 (70)	143/178 (80)	2/178 (1)
Hahella chejuensis<br>YP_433029.1 KTC 2396	Ni,Fe-hydrogenase I small subunit	85/178 (48)	116/178 (65)	12/178 (7)
Agarivorans albus<br>WP_016400415.1 MKT 106	Secreted trypsin-like serine protease	85/178 (48)	104/178 (58)	18/178 (10)
Homology across conserved peptidase motif				
Alteromonas macleodii<br>YP_006749171.1 ATCC27126	Hypothetical protein	16/22 (73)	17/22 (77)	0/22 (0)
Aeromonas hydrophila<br>YP_855156.1 ML09-119	Hypothetical protein	14/22 (64)	14/22 (64)	0/22 (0)
Aeromonas aquariorum<br>WP_010635185.1	Hypothetical protein	14/22 (64)	14/22 (64)	0/22 (0)
Vibrio cholerae O1<br>WP_000710201.1	Hypothetical protein	14/22 (64)	17/22 (77)	0/22 (0)
Vibrio mimicus<br>WP_000710197.1 VM543	Hypothetical protein	14/22 (64)	16/22 (73)	0/22 (0)
Vibrio vulnificus<br>WP_002810048.1 MO6-24/O	Hypothetical protein	14/22 (64)	17/22 (77)	0/22 (0)
Xanthomonas fragariae<br>WP_002810048.1 LMG 25863	Hypothetical protein	12/22 (54)	13/22 (59)	2/22 (9)
Xanthomonas campestris<br>M72.UPW<br>WP_010375553.1	Peptidyl-Asp metalloendopeptidase	12/22 (54)	13/22 (59)	2/22 (9)
Xanthomonas vesicatoria<br>M72.UPW<br>WP_005991741.1	Peptidyl-Asp metalloendopeptidase	12/22 (54)	13/22 (59)	2/22 (9)
