# Supplementary material for: Characterization of the Pseudomonas aeruginosa metalloendopeptidase, Mep72, a member of the Vfr regulon
Source: BMC Microbiol. 2013 Nov 27;13:269. doi: 10.1186/1471-2180-13-269 (PMC4222646; doi:10.1186/1471-2180-13-269)
Supplement: Additional file 3 — The predicted PA2783 protein carries two carbohydrate-binding modules. Interrogation of the non-redundant databases at NCBI (http://www.ncbi.nlm.nih.gov/; accessed 06/19/2013) using BLASTP revealed homology with the CBM_4_9 family (Cdd:pfam02018) of diverse CHO-binding proteins. CHO-binding domain I (A) and domain II (B), have different aa sequences but both were strongly homologous to the two CHO-binding modules of the Pseudomonas mendocina (Pmendo) carbohydrate-binding CenC domain-containing protein and the Ni,Fe-hydrogenase I small subunit of Hahella chejuensis (Hcheju). For the pfam, identical aa are indicated by * and similar aa by ^; for bacterial proteins, identical aa are shown in red, similar aa in blue, and non-similar aa in black; Pmucil, Paenibacillus mucilaginosus; Clen-1 and Clen-2, Cellulosilyticum lentocellum CHO-binding domains I and II. Percentages of aa identity and similarity are shown in Additional file 4. [file 1471-2180-13-269-S3.pdf]

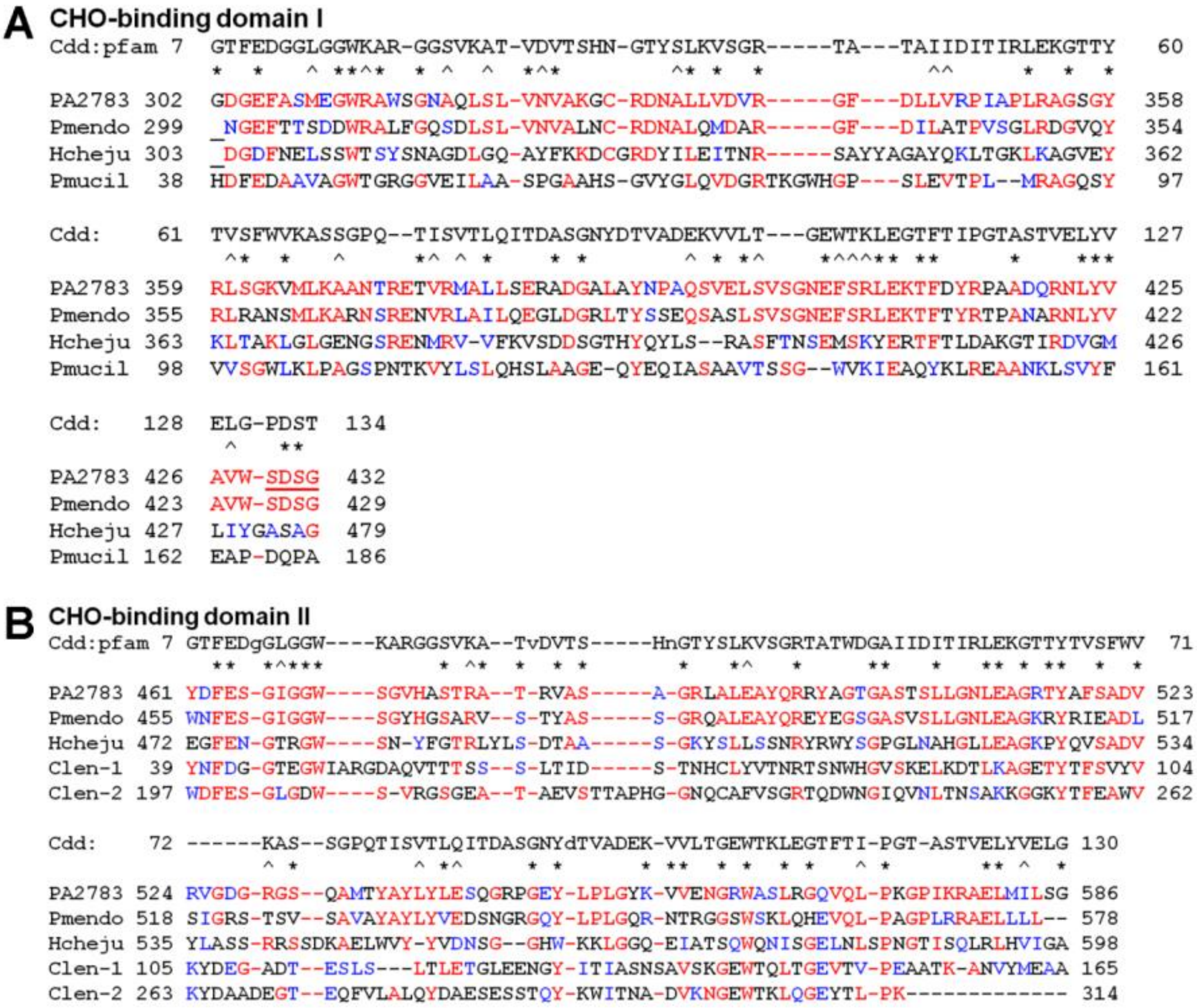

Additional file 3 - The predicted PA2783 protein carries two carbohydrate-binding modules

Interrogation of the non-redundant databases at NCBI (http://www.ncbi.nlm.nih.gov/; accessed 06/19/2013) using BLASTP revealed homology with the CBM_4_9 family (Cdd:pfam02018) of diverse CHO-binding proteins. CHO-binding domain I **(A)** and domain II **(B)**, have different aa sequences but both were strongly homologous to the two CHO-binding modules of the *Pseudomonas mendocina* (Pmendo) carbohydrate-binding CenC domain-containing protein and the Ni,Fe-hydrogenase I small subunit of *Hahella chejuensis* (Hcheju). For the pfam, identical aa are indicated by * and similar aa by ^; for bacterial proteins, identical aa are shown in red, similar aa in blue, and non-similar aa in black; Pmucil, *Paenibacillus mucilaginosus*; Clen-1 and Clen-2, *Cellulosilyticum lentocellum* CHO-binding domains I and II. Percentages of aa identity and similarity are shown in Additional file 4.
