# Supplementary material for: Characterization of the Pseudomonas aeruginosa metalloendopeptidase, Mep72, a member of the Vfr regulon
Source: BMC Microbiol. 2013 Nov 27;13:269. doi: 10.1186/1471-2180-13-269 (PMC4222646; doi:10.1186/1471-2180-13-269)
Supplement: Additional file 4 — Amino acid homology of the predicted PA2783 protein carbohydrate-binding domains I and II with other bacterial proteins. [file 1471-2180-13-269-S4.pdf]

Additional file 4 - Amino acid homology of the predicted PA2783 protein carbohydrate-binding domains I and II with other bacterial proteins

Organism<br>Accession Number	Protein	Identity (%)	Similarity (%)	Gaps (%)
	CHO-binding module domain I			
Pseudomonas mendocina<br>YP_001189641.1 ymp	CHO-binding CenC domain-containing protein	80/131 (60)	93/131 (71)	1/131 (0.7)
Hahella chejuensis<br>YP_433029.1 KTC 2396	Ni,Fe-hydrogenase I small subunit	35/131 (27)	60/131 (46)	4/131 (3)
Paenibacillus mucilaginosus<br>YP_006190919.1 K02	Endo-1,4-beta-xylanase	37/131 (27)	60/131 (46)	4/131 (3)
Pseudomonas fluorescens<br>WP_003193107.1 SS101	Copper oxidase	38/131 (29)	59/31 (45)	16/102 (12)
**Cdd: CBM_4_9 pfam02018**	CHO-binding module	33/131 (25)	51/131 (39)	6/131 (5)
	CHO-binding module domain II			
Pseudomonas mendocina<br>YP_001189641.1	CHO-binding CenC domain-containing protein	69/126 (55)	87/126 (69)	2/126 (1.5)
Hahella chejuensis<br>YP_433029.1	Ni,Fe-hydrogenase I small subunit	38/126 (30)	62/126 (49)	7/126 (5.5)
*Cellulosilyticum lentocellum**<br>YP_004307454.1	Endo-1,4-beta-xylanase (aa 34-173)	32/126 (25)	53/126 (42)	9/126 (7)
*Cellulosilyticum lentocellum**<br>YP_004307454.1	Endo-1,4-beta-xylanase (aa 190-314)	34/126 (27)	46/126 (36)	20/126 (16)
**Cdd: CBM_4_9 pfam02018**	CHO-binding module	39/126 (31)	47/126 (37)	11/126 (9)

*Previously *Clostridium lentocellum*
